# Variation in the Dispersions of Powder Liquid Ratios in Hand-Mix Glass Ionomers

**DOI:** 10.2174/1745017901814010647

**Published:** 2018-09-28

**Authors:** Riaan Mulder

**Affiliations:** Pediatric Dentistry Department, Faculty of Dentistry, University of the Western Cape, Private Bag X1, Tygerberg, 7505 Cape Town, South Africa

**Keywords:** Hand-mix, Powder/liquid ratio, Glass ionomer, Liquid weight, Powder weight, Manufacturer’s recommendation

## Abstract

**Background:**

The Powder/Liquid Ratio (PLR) influence, and the literature regarding the handling and physical properties of Glass Ionomer restorative materials (GIC) were investigated.

**Objective:**

The objective of the study was to compare the PLR variability and magnitude in hand-mix GICs, as dispensed for clinical use. From the recorded individual powder and liquid weights, additional comparisons could be made by pairing the various “extreme” outer observations in relation to the manufacturer’s PLR.

**Study Design:**

The materials assessed were Ketac Universal Hand-mix (KUH), Riva Self Cure Hand-mix (RSCH) and Fuji IX GP Hand-mix (FIXH). Twenty scoops of powder were paired with twenty drops of liquid, as would be the case in the clinical scenario. Statistical analysis was completed with the Kruskal Wallis H test, Intraclass Correlation (ICC) and straight line regressions with One-way ANOVA and the post-hoc Tukey HSD Test (*p*<0.05 was considered significant).

**Results:**

The powder and liquid observations indicate a lack of consistency in both the powder and liquid dispersions. The volume remained “one drop” but the weights were much lower than the manufacturer’s recommended drop weight for some observations, due to air in the liquid drop. The Kruskal-Wallis H test indicated significant differences (*p*=0.0001) between the three materials for the paired PLRs. The One-way ANOVA and post-hoc Tukey HSD Test were used to compare the recommended PLR to the results and the significant differences (*p*<0.01).

**Conclusion:**

The recommended manufacturers’ powder and liquid weights were KUH 0.150/0.05g; RSC 0.165/0.035; FIXH 0.18/0.05, respectively. KUH, FIXH and RSCH liquid had powder and liquid dispersions above the manufacturer’s recommendations. FIXH had the most paired PLR observations within the ±10% range followed by KUH. Extreme powder and liquid combinations could occur in the clinical scenario and these combinations were considered.

## 1 INTRODUCTION

The literature shows that Capsulated Glass Ionomer restorative materials (GIC) stay superior to the hand-mixed versions with regard to the consistent mixing regime and physical properties [1, 2]. The capsulated GIC Powder/Liquid Ratio (PLR) is not influenced as severely as the variation in PLR of hand-mix GIC [1]. Besides an increased fluoride release [3] the capsules have a definitive advantage due to the decrease in operator variability [2] and increased ease of application in the cavity preparation [4]. The clinician induced variation in PLR in relation to the manufacturer’s recommendation, results in the compressive and diametral tensile strength being influenced [5]. The reduction in the physical properties also has clinical relevance with regard to the wear resistance. Hand-mix GICs are more regularly used in clinical practice compared to capsulated GIC’s. The clinical manipulations of hand-mix GICs by clinicians are based on personal preference of the material consistency [6]. This preferred consistency does not necessarily reflect the manufacturer’s PLR recommendation, with the speed of spatulation and the powder content being manipulated by clinicians [5, 6]. Clinician variability markedly reduces the powder content of GIC to below the manufacturer’s recommendation to as much as -50%. Therefore, when this reduction in the PLR range is considered when anterior teeth are restored, the capsulated GICs are a suitable alternative [7].

The hypothesis was that the powder and the liquid dispensed would be the same as the manufacturer’s recommendation. The aim of this study was to assess the PLR variability and the magnitude in hand-mix GICs, as dispensed for clinical use. From the recorded individual powder and liquid weights, additional comparisons could be made by pairing the various “extreme” outer observations in relation to the manufacturer’s PLR.

## 2 MATERIALS AND METHODS

This *in vitro* comparative study of three GIC hand-mix materials was completed in accordance with the manufacturer’s instructions.

### 2.1 Specimen Preparation

Three GIC materials were used (Table **1**). A total of 20 powder scoops and 20 liquid drops from the materials were dispensed (n=60 powder, n=60 liquid drops). The researcher dispensing the powder and the liquid was blinded by the research assistant and the materials randomly given to the researcher for assessment. The researcher performed the weight determination of the powder, followed by the liquid at a constant room temperature (23 ± 1°C) with a relative humidity of 50 ± 5% [8]. The weight determination of the powder and the liquid were completed on a desktop chemical scale to an accuracy of 0.0001 g *(Metler AE240 analytical balance, Columbus, Ohio, USA).*

### 2.2 Test Parameters

One scoop of powder and one drop of liquid was dispensed under standardised laboratory conditions to be “paired” as an observation for the PLR. The powder and liquid pairing was done in order to calculate the PLR as would have been the case in the clinical scenario.

### 2.3 Statistical Analysis

The observed powder and liquid values were paired as initially dispensed. These results were assessed with the Kruskal Wallis H test, Intraclass Correlation (ICC) and straight line regressions. The twenty-paired observations were firstly compared with a Kruskal-Wallis H test to determine if there were differences in the PLR score between the three groups: Ketac Universal Hand-mix (KUH), Riva Self Cure Hand-mix (RSCH) and Fuji IX GP Hand-mix (FIXH). In order to determine the difference and correlation with the manufacturer’s PLR recommendation, an intraclass correlation was done with the two-way mixed-effect model at a 95% confidence interval (*p*<0.05 as significant).

The values obtained in this observational study were further compared in the format of the percentage difference between the manufacturer’s PLR recommendation and the observed dispension of powder and liquid. A graphical representation of the straight-line regression of the observations was made with a ±10% limit from the observed value as well at the manufacturer’s recommendation. The PLR of the manufacturer was compared with the PLR of this observational study. The statistical analysis was completed after the straight-line regression with One-way ANOVA and the post-hoc Tukey HSD Test (Statistical analysis with R Core Team (2013); (R: A language and environment for statistical computing. R Foundation for Statistical Computing, Vienna, Austria).

## 3 RESULTS

The Kruskal-Wallis H test indicated the differences in the paired PLR magnitude between the three materials. Mean PLR scores had statistically significant differences between materials, *p* = 0.0001.

No degree of reliability was found with the Intraclass Correlation (ICC) between the paired PLR ratio and the manufacturers' recommended PLR for KUH, RSCH nor FUIX. The confidence interval included zero and the *p*>0.05, the ICC was not regarded as statistically significant.

The manufacturers’ recommended PLRs are represented in Table ****1****. Fig. (**1**) represents the observations of the powder, liquid and the recommended PLR of the three manufacturer’s PLR. The horizontal lines indicate the ±10% allowable variation in order to retain the physical properties of the GIC Fig. (**1**). The graphs illustrate that all the paired KUH and FIXH ratios are greater than the manufacturer’s recommended PLR. The opposite was found for the RSCH ratios.

**Fig. (1):**
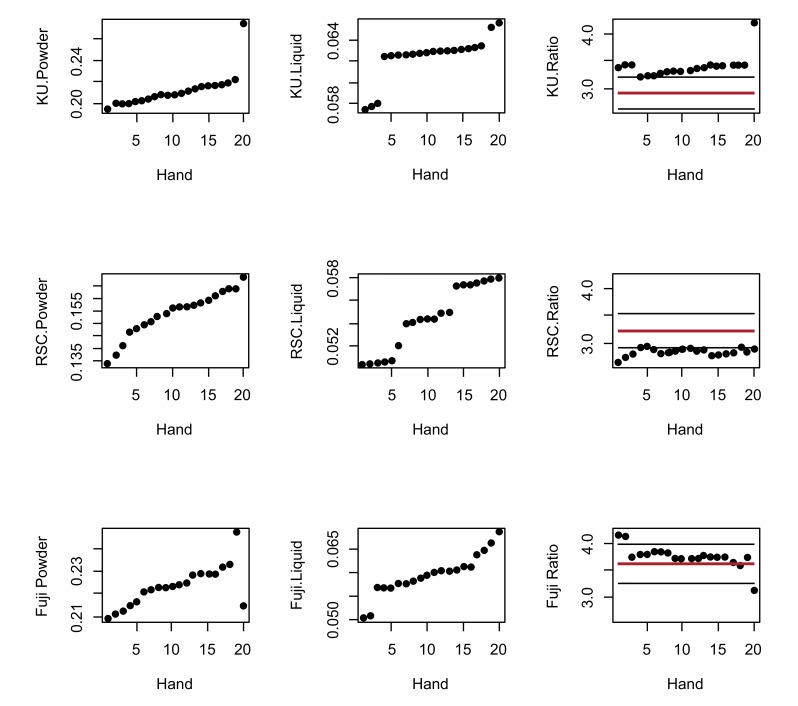
Powder, liquid and PLR observations.

The powder and liquid observations shown in the graph indicate the lack of consistency from both the powder and liquid dispensing (Fig. **1**). The trend from the first 19 observations is nearly linear with random variations. The standard deviation obtained from a straight-line regression is 0.00111. According to this regression, the expected value at observation number 20 should have been 0.2227. This means that the observed KU powder observation number 20 was 0.275, resulting in a difference from the expected value of (0.275-0.2227) / 0.00111 = 47.1 standard deviations. This illustrates that the KUH powder observation number 20 can be considered an outlier.

Based on the random error standard deviation for FIXH, the successive difference of the first 18 observations was 0.00134. The estimated standard deviation of the difference between two observations for FIXH is 0.00134√2=0.00190. The observed difference between the observations 18 and 19 was 0.0139, *i.e*. 7.3 standard deviations, denoting 18 and 19 outliers. Similarly, the difference between FIXH observation 20 and 19 was 0.0324, with 17.1 standard deviations.

It was therefore applicable to perform a comparison between the manufacturers’ PLR recommendation *versus* the twenty-paired PLR observations. The One-way ANOVA and post-hoc Tukey HSD Tests indicated significant differences with *p*<0.01 between the KUH, FIXH and RSCH manufacturer’s PLR in relation to the PLR of each glass ionomer assessed respectively.

Table. **2** illustrates the individual observations as they were paired: one powder to be mixed with one liquid, as would be the case for the clinical scenario. The paired observations from KUH, FIXH and the RSCH liquid had observations above the +10% mark. KUH and FIXH had the most paired PLR observations for both powder and liquid observations above the +10% ratio in relation to the manufacturer’s recommendation. Although the paired powder and liquid weights were different from the manufacturer’s recommended PLR of each scoop or drop, the final PLR remained within the ±10% range of the manufacturer’s recommendation for FIXH [17] and KUH [7]. RSCH showed all 20 paired observations below the -10% PLR of the manufacturer’s recommendation even though the RSCH powder had 15 of the observations within the ±10% of the manufacturer’s recommendation.

Table **3** shows the various combinations of the smallest and largest values of the powder and the liquid observations. Although the 20 powder observations were paired with the 20 liquid observations in Table **2** for statistical purposes, in order to simulate the clinical scenario other “extreme” combinations are possible. It was plausible to consider that any combination of the powder and liquid observations could have occurred in the clinical scenario. The variation of the PLR in the dispensing stage can occur due to the variation of the powder and/or the liquid. Table. **3** illustrates the extremes of PLR variation, *e.g*. largest powder in combination with smallest liquid observation.

For the most part, the PLR percentage difference in Table **3** illustrates that all three manufacturers had variations from the recommended PLR when the smallest and largest combinations were evaluated. RSCH PLR combinations of the powder and liquid had all the extreme combinations of the PLR well below the manufacturer’s recommendation (Table **3**). The comparison of the extreme PLR values for KUH and FIXH had PLR both above and below the manufacturer’s PLR. The percentage difference between the various extreme values ranged between 22.11% (RSCH), 33.97% (FUIXH) and 42.08% (KUH) (Table **3**). For RSCH, on the other hand, the value of the liquid being above the +10% mark and the powder within or below the manufacturer’s recommendation, resulted in the PLR in Table **3** being well below the manufacturer’s PLR recommendation. FIXH, interestingly had values above the +10% for the powder and the liquid (Table **2**), but the PLR presented values either above or below the manufacturer’s PLR (Table **3**). Based on the results, the hypothesis that the powder and the liquid dispensed would be the same as the manufacturer’s recommendation, was rejected.

## 4 DISCUSSION

The powder densities determine the weight per volume of the powder present in the scoop [1, 5, 9, 10]. The hygroscopic nature of the powder was not relevant for this study, since the material seals were broken at the commencement of the study and the observations immediately completed. The temperature, angle and the finger pressure applied to the bottle during liquid dispensing play a role in the amount of liquid dispensed [1, 5, 9, 10]. It was noted that air bubbles were present in some drops of liquid from all manufacturers. The volume remained “one drop” but the weight was much lower than the manufacturer’s recommended drop weight. These are all factors where the powder and liquid weight play a role and therefore influence the handling and physical properties of the materials.

Compressive strengths obtained by variation in the PLR of hand-mix GIC indicated that a 100% PLR ratio was not significantly different from a 90% PLR ratio, but significantly different from 80%. An 80% PLR ratio (20% less than the manufacturer’s recommendation) also resulted in statistically significant longer setting times, as less powder was incorporated into the constant volume of liquid [1]. For this reason, 10% less than the manufacturer’s recommendation is acceptable as a PLR variation. A decreased PLR affects the physical properties and increases the setting time [11]. The decrease in filler particles leads to a weaker material. With GICs being more active in the presence of an acidic environment [12], the decrease of the pH will result in an increased acid erosion of the restoration [13]. This poses a problem, considering that the higher caries risk patient is specifically indicated for a GIC. The Vickers Hardness (VH) and the three body wear tests for a GIC luting cement illustrated that when the GIC liquid content increased 17% more than the manufacturer’s recommended PLR, there was a 50% decrease in both the VH and wear resistance of the GIC luting cement. The increase of the powder 17% above the manufacturer’s recommendation did not negatively affect the VH and the wear resistance. The increase of the liquid more than the aforementioned 17% resulted in samples that could not withstand wear cycles [14]. The higher the amount of powder, the greater are the mechanical properties of GICs [15]. The powder amount correlates with the compressive strength and wear resistance. The solubility, setting and working time is indirectly correlated with the amount of powder [1, 16, 17].

The powder and liquid dispensing prior to mixing should be aimed at being as close to the manufacturer’s recommendation as possible to prevent change in the physical properties. If clinicians are made aware of the impact of variation from the recommended PLR, one might find a greater acceptance of GIC materials. A study performed with a hand-mixed GIC, assessed the physical properties with various powder ratios to a constant liquid ratio that was maintained at 1g. This study found that powder ratios 50% and 80% less than the manufacturer instructions statistically decreased the compressive strength as well as the setting time of the GIC tested [1]. This decrease in powder lead to a decreased concentration of reinforcing glass particles, which resulted in a decreased load bearing capacity. The clinician should attempt to mix GICs with as high a powder content as possible, provided that there is still enough liquid for the gelation to occur. This will translate well for the RSCH materials that were well below the -10% value for the PLR extremes. GICs with a high powder content would be more resistant to the effects of moisture, have a shorter working time and a higher compressive strength. The mixed material will be a clinically visible viscous paste [17]. The largest powder and the smallest liquid ratio should result in a higher PLR. It was noted that the extreme ranges of KU (+40.71%) and FIXH (+18.56%) had values well above the +10% range. RSC extreme ranges still obtained a -28.91% below the recommended PLR of the manufacturer. If the powder ratio was more than what the liquid can effectively hydrate, a faster setting reaction will take place and the material will set before the restoration is placed in the prepared cavity [1]. The variability in the liquid to powder ratio will affect the clinical handling and physical properties of the GIC [13]. In general, FUIXH seemed to have been the closest to the manufacturer’s recommendation, although the ICC was not significant. This could explain the Fuji IX hand-mix GICs that performed better than the capsulated version in a previous study from 2007 [18].

Fuji IX hand-mix and Fuji IX Fast Capsules have the same PLR according to the manufacturer’s recommendation. At the manufacturer’s PLR recommendations the hand-mix Fuji IX had a significantly higher compressive strength compared to the Fuji IX Fast Capsule group. The 40 second (s) hand-mix was longer than the 10s (3M ESPE, Capmix^TM^) or 8s with 3s of centrifuging (3M ESPE, Rotomix^TM^) in accordance with the manufacturers’ instructions. The short capsule titration *vs* the 40s hand-mix was suggested as the reason why the hand-mix could have had more homogeneity for the powder and liquid mix [19].

## CONCLUSION

The increase in powder above the manufacturer’s recommendation with a constant liquid will result in a PLR above the manufacturer’s recommendation. Increased setting time and increased physical properties will occur. The ideal would be to have a consistent dispersion of powder and liquid in the clinical scenario. The irregularity in dispersions has contributed to the possibility of various extreme PLR combinations. The variation due to air bubbles in the liquid upon dispensing was not a significant contributor to variations in PLR. Increasing the scoop size per dispension could be a strategy to mitigate the low PLR for RSCH. Although the FIXH powder and liquid weights were greater than the recommended manufacturer’s PLR recommendation, the observations from FIXH were the closest to the manufacturer recommendation. The reality is that extreme powder and liquid combinations could occur in the clinical scenario. The clinical implications are that too little liquid with too much powder will also have an increased PLR. Too much powder, or too little liquid will result in an insufficient acid-base reaction with a decrease in the physical properties, change in the working time and an altered manipulation ability of the clinician during restoration placement.

## Figures and Tables

**Table 1:** Manufacturer supplied recommendation.

**Material and Manufacturer**	**Material Abbreviation**	**Recommended Powder (g)**	**Manufacturer Recommended Liquid (g)**	**Manufacturer Recommended PLR**
Ketac Universal hand-mix (3M ESPE, Seefeld, Germany). Batch: 583514.	KUH	0.150	0.05	3:1
Riva Self Cure hand-mix (SDI Limited, Australia). Batch: 62657V.	RSCH	0.165	0.035	4.7:1
Fuji IX GP hand-mix (GC Corp, Tokyo, Japan). Batch: 1503231.	FIXH	0.18	0.05	3.6:1

**Table 2:** Paired observations distribution between ±10% limits of the manufacturer’s recommendation.

**Material**	**Observations within ±10%**	**Observations below -10%**	**Observations above +10%**	**Paired Observation PLR within ±10%**	**Paired Observation PLR below -10%**	**Paired Observation PLR above +10%**
KUH Powder	0	0	20	7	0	13
KUH Liquid	0	0	20			
RSCH Powder	15	5	0	0	20	0
RSCH Liquid	0	0	20			
FIXH Powder	0	0	20	17	1	2
FIXH Liquid	2	0	18			

**Table 3:** Powder liquid combinations from observations.

**Material**	**“Extreme” Observations**				
	**Mean P or Mean L**	**Highest P or Highest L**	**Lowest P or Lowest L**	**Highest P or Lowest L**	**Lowest P or Highest L**
KUH Powder	0.2119^+^	0.275^+^	0.1938^+^	0.275^+^	0.1938^+^
KUH Liquid	0.0623^+^	0.0655^+^	0.0575^+^	0.0575^+^	0.0655^+^
KUH PLR	3.3987:1^+^	4.1984:1^+^	3.3704:1^+^	4.7826:1^+^	2.9587:1
KUH PLR % different to manufacturer’s recommendation	+13.29%	+39.94%	+12.34%	+40.71%	-1.37%
RSCH Powder	0.15384	0.1684	0.1335^-^	0.1684	0.1335^-^
RSCL Liquid	0.05446^+^	0.058^+^	0.0504^+^	0.0504^+^	0.058^+^
RSC PLR	2.8249:1^-^	2.9034:1^-^	2.6488:1^-^	3.3412:1^-^	2.3017:1^-^
RSCH PLR % different to manufacturer’s recommendation	-39.89%	-38.22%	-43.64%	-28.91%	-51.02%
FIXH Powder	0.223165^+^	0.2147^+^	0.2089^+^	0.2147^+^	0.2089^+^
FIXH Liquid	0.05958^+^	0.0686^+^	0.0503^+^	0.0503^+^	0.0686^+^
FIXH PLR	3.7456:1	3.1297:1^-^	4.153:1^+^	4.2683:1^+^	3.0451:1^-^
FIXH PLR % different to manufacturer’s recommendation	+1.04%	-13.06%	+15.36%	+18.56%	-15.41%
The “+” indicate a value above +10% and “-“ below -10%, of the manufacturer powder weight per scoop or liquid weight per drop recommendation in Table **1**.					

## LIST OF ABBREVIATIONS

FIXH: = Fuji IX GP Hand-mix
GIC: = Glass Ionomer Restorative material
ICC: = Intraclass Correlation
KUH: = Ketac Universal Hand-mix
PLR: = Powder Liquid Ratio
RSCH: = Riva Self Cure Hand-mix
